# Induction of immunogenic cell death in radiation-resistant breast cancer stem cells by repurposing anti-alcoholism drug disulfiram

**DOI:** 10.1186/s12964-019-0507-3

**Published:** 2020-03-05

**Authors:** Ting Sun, Wei Yang, Sneh M. Toprani, Wei Guo, Lile He, Albert B. DeLeo, Soldano Ferrone, Gong Zhang, Enwen Wang, Zunwen Lin, Pan Hu, Xinhui Wang

**Affiliations:** 1 Division of Surgical Oncology, Department of Surgery, Massachusetts General Hospital, Harvard Medical School, Boston, USA; 2 Neurosurgery and Brain and Nerve Research Laboratory, The First Affiliated Hospital of Soochow University, Suzhou, Jiangsu China; 3 John B. Little Center for Radiation Sciences, Harvard T.H. Chan School of Public Health, Boston, USA; 4 State Key Laboratory of Radiation Medicine and Protection, School of Radiation Medicine and Protection and Collaborative Innovation Center of Radiation Medicine of Jiangsu Higher Education Institutions, Soochow University, Suzhou, China; 5 Department of Orthopaedic Surgery, Massachusetts General Hospital, Harvard Medical School, Boston, USA

**Keywords:** Breast cancer, Stem cells, Immunogenic cell death, Radiation, Disulfiram, Copper, Reactive oxygen species, IRE1α, XBP1s, Signaling pathway

## Abstract

**Background:**

The current successful clinical use of agents promoting robust anti-tumor immunity in cancer patients warrants noting that radiation therapy (RT) induces immunogenic cell death (ICD) of tumor cells, which can generate anti-tumor immune responses. However, breast cancer stem cells (BCSCs) are resistant to RT and RT alone usually failed to mount an anti-tumor immune response.

**Methods:**

High aldehyde dehydrogenase activity (ALDH)^bright^ and CD44^+^/CD24^−^/ESA^+^ cancer cells, previously shown to have BCSC properties, were isolated from human MDA-MB-231 and UACC-812 breast cancer cell lines by flow cytometer. Flow sorted BCSCs and non-BCSCs were further tested for their characteristic of stemness by mammosphere formation assay. Induction of ICD in BCSCs vs. non-BCSCs in response to different in vitro treatments was determined by assessing cell apoptosis and a panel of damage-associated molecular pattern molecules (DAMPs) by flow and enzyme-linked immunosorbent assay (ELISA).

**Results:**

We found that ionizing radiation (IR) triggered a lower level of ICD in BCSCs than non-BCSCs. We then investigated the ability of disulfiram/cooper (DSF/Cu) which is known to preferentially induce cancer stem cells (CSCs) apoptosis to enhance IR-induced ICD of BCSCs. The results indicate that DSF/Cu induced a similar extent of IDC in both BCSCs and non-BCSCs and rendered IR-resistant BCSCs as sensitive as non-BCSCs to IR-induced ICD. IR and DSF/Cu induced ICD of BCSCs could be partly reversed by pre-treatment of BCSCs with a reactive oxygen species (ROS) scavenger and XBP1s inhibitors.

**Conclusion:**

DSF/Cu rendered IR-resistant BCSCs as sensitive as non-BCSCs to IR-induced ICD. Our data demonstrate the potential of IR and DSF/Cu to induce ICD in BCSCs and non-BCSCs leading to robust immune responses against not only differentiated/differentiating breast cancer cells but also BCSCs, the root cause of cancer formation, progression and metastasis.

**Graphical abstract:**

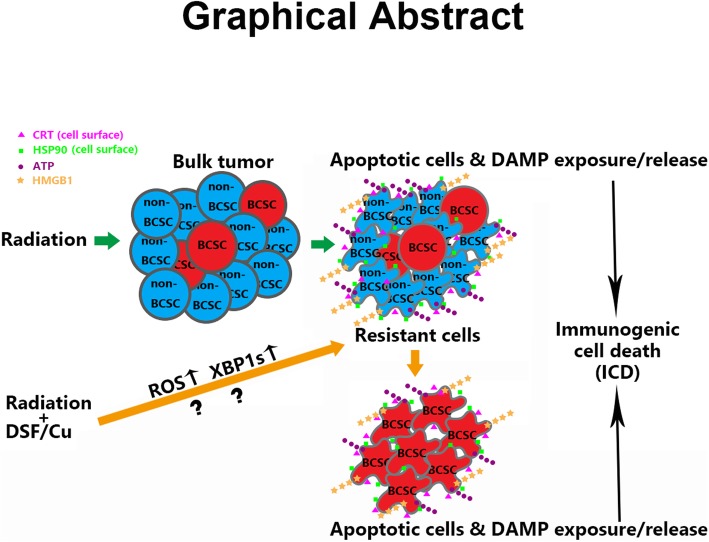

## Background

Breast cancer is the most common cancer among women. The development of more effective therapeutic regimens has contributed to improved outcomes for breast cancer patients. Nevertheless, recurrence and metastasis often happen after comprehensive treatments. Emerging evidence indicates that preexisting CSCs, a small subpopulation of cancer cells that exist within heterogeneous tumors, are responsible for treatment resistance and subsequent progression, recurrence, and metastasis of cancer [1–4].

CSCs express two fundamental characteristics: the capacity for self-renewal and the ability to efficiently reconstitute differentiated tumors. In contrast, non-stem cancer cells, the differentiated/ differentiating progeny that constitute a substantial part of the tumor, are radiosensitive, which accounts for the short-term regression of tumors following RT.

Tumor cells expressing elevated levels of intracellular aldehyde dehydrogenase (ALDH) in human and mouse breast cancer have been convincingly shown to be BCSCs [5, 6]. The enhanced ALDH activity in these cells eliminates genotoxic aldehydes contributing to their resistance to most standard cancer therapies. CSC research is facilitated by the ability to detect and isolate these cells by flow cytometry using the ALDEFLUOR reagent to measure ALDH enzymatic activity [6, 7]. ALDH^bright^ cells are defined as those ALDH^+^ cells with twice the mean fluorescence intensity (MFI) of the bulk ALDH^+^ cell population.

DSF is an irreversible pan-ALDH inhibitor [8, 9] and FDA approved drug for treatment of alcoholism since 1951 [10]. It’s uses results in accumulation of, in particular, acetaldehyde, and unpleasant effects in individuals when they consume alcohol. Interestingly and strikingly, an epidemiological study covering a 13-year period indicated that treatment of alcoholism in cancer patients with DSF significantly reduced their risk of death from cancer [11].

Studies have shown that in cells DSF converts to diethyldithiocarbamate (deDTC) and that two molecules of deDTC bind to one molecule of copper (Cu^2+^) to form the Cu [deDTC] complex (DSF/Cu) [12–14]. It is known that DSF/Cu is an effective proteasome inhibitor resulting in inhibition of NF-κB [12, 15] and activation of endoplasmic reticulum (ER)-stress through upregulation of the inositol requiring-enzyme 1 alpha (IRE1α)- X-box-binding protein 1 (XBP1) axis leading to autophagic apoptosis [16]. It was demonstrated that DSF/Cu is a potent radio- and chemo-sensitizer for breast and pancreatic cancer through targeting CSCs [6, 17].

IR has been reported as a strategy to induce ICD in a variety of tumor cells [18–23]. The phenomenon of ICD, the emission of immuno-stimulatory signals by dying apoptotic cells, is induced by chemotherapeutic drugs such as anthracyclines, oxaliplatin, bortezomib, or radiotherapy and photodynamic therapy [24–26] can lead to effective antitumor immune responses [27–31]. The molecular characteristics of ICD are referred to as the release or cell-surface expression of highly immunostimulatory DAMPs [32] such as i) the surface exposure of calreticulin (CRT) and heat shock proteins (HSPs), ii) extracellular secretion of adenosine triphosphate (ATP), and iii) passive release of high mobility group protein B1 (HMGB1). These molecules stimulate antigen presenting cells (APCs) and activate dendritic cells can lead to the subsequent development and activation of tumor-specific effector T cells and memory T cells [32].

The results of an investigation of ICD of BCSCs and non-BCSCs induced by radiation and/or DSF/Cu are reported here. BCSCs present in two well-established human breast cancer cell lines, MDA-MB-231 and UACC-812, identified and isolated by flow cytometry as either ALDH^bright^ or CD44^+^/CD24^−^/ESA^+^ cells [5, 6, 33] were used in this study. Induction of ICD of both types of BCSC populations and non-BCSCs by radiation and DSF/Cu was monitored by apoptosis and expression/release of DAMPs. In addition, the roles of intracellular ROS and the IRE1α/XBP1s pathway, both of which are involved in DSF/Cu induced apoptosis of breast cancer cells [15, 16] in inducing ICD in BCSCs was studied.

## Materials and methods

### Cell culture

Human breast cancer cell lines, MDA-MB-231 and UACC-812 were obtained from the Duke Comprehensive Cancer Center Cell Culture Facility and the American Type Culture Collection (ATCC), respectively. MDA-MB-231 cells were cultured in high glucose DMEM (Corning Incorporated, NY, USA), and UACC-812 cells were cultured in RPMI 1640 (Corning) with 10% fetal bovine calf serum (FBS) (Gemini Bio-Products, CA, USA) at 37 °C in a humidified atmosphere of 5% CO_2_. Sorted cells were seeded and incubated overnight in 24-well plates at density of 5 x 10^4^/well in 0.5 mL of the same medium with 20% FBS and 1% penicillin and streptomycin (Corning).

### Fluorescence-activated cell sorting (FACS)

To sort the BCSC subpopulations in the human breast cancer MDA-MB-231 and UACC-812 cell lines, 5–10 x 10^7^ cells were used at a time. ALDH assay kit from STEMCELL Technologies Inc. (Vancouver, BC, Canada), the antibodies for cell surface markers CD44, CD24 and ESA were FITC- conjugated anti-human CD44 antibody (clone BJ18), PE conjugated anti-human CD24 antibody (clone ML5), APC conjugated anti-human ESA (EpCAM) antibody (clone 9C4) from Biolegend (Dedham, MA, USA) were used to stain cells. For sorting ALDH^bright^ cells, cells were incubated with ALDEFLUOR® reagent with or without ALDH inhibitor N,N-diethylaminobenzaldehyde (DEAB) (STEMCELL) at 37 °C for 1 h according to the manufacturer’s instructions. For sorting CD44^+^/CD24^−^/ESA^+^ cells, cells were incubated with the 3-antibody cocktail at 4 °C for 15 min in the dark. Dead cells were excluded by 7-AAD staining. Stained cells were analyzed by FACSAria II and sorted by BD FACSAria I sorters at HSCI-CRM Flow Cytometry Core Facility, Massachusetts General Hospital.

### Real-time quantitative reverse transcription PCR (qRT-PCR)

Sorted ALDH^bright^ and ALDH^dim^ cells were used for SYBR Green -based qRT-PCR to detect the mRNA levels of ALDH1A1 with primers (forward, 5′-GGAGGAAACCCTGCCTCTTTT-3′ and reverse, 5′-TTGGAAGATAGGGCCTGCAC-3′) as described [34, 35].

### Mammosphere formation assay

Sorted cells (1 × 10^3^ cells) were seeded in a 24-well ultra-low adherent plate (Corning) in 0.5 mL of mixed medium to perform sphere formation detection. The medium contained 32% MethoCult medium, 20% MammoCult basal human medium with a final concentration of 2% MammoCult proliferation supplements and 48% DMEM supplemented with final concentrations of 100 pg/mL EGF, 50 ng/mL bFGF, 5 ng/mL stem cell factor, 1 μM hydrocortisone, and 5 mg/mL insulin, all obtained from STEMCELL Technologies. The cells were cultured at 37 °C in a 1% O_2_ and 5% CO_2_ humidified atmosphere for 14 d. The number of tumor spheres was counted under microscope.

### Chemical reagents, in vitro cell treatment and radiation

Tetraethylthiuram disulfide (disulfiram, DSF) and Copper (II) chloride (Cu) were purchased from Sigma-Aldrich. DSF was dissolved in DMSO, and Cu was dissolved in Milli-Q water. The sorted cells were treated with DSF/Cu in the concentration of 0.15/1 μM. This low dose was chosen based on titration experiments in order to obtain sufficient cells for the subsequent experimental analyses. IR was performed with single doses of 8 Gy or 12 Gy. The X-RAD 320 Biological Irradiator (Precision X-ray, Inc., CT, USA) was used for IR experiments in this study.

### Flow cytometric analyses for apoptotic cells, cell surface expression of CRT and HSP90

Apoptotic cells were identified using the Annexin V/Dead Cell Apoptosis Kit (Biolegend) according to the manufacturer’s instructions. The adherent cells were trypsinized, washed with PBS and resuspended in the binding buffer included in the Kit, then stained with FITC–Annexin V and 7-AAD for 15 min at 4 °C in the dark. Cell surface proteins were detected using a two-step method. Cells were collected and washed twice with PBS before being stained. Cells were incubated with primary antibodies respectively including mouse anti-human CRT antibody TO-11 [36] and human anti-human HSP90 antibody (W9 which recognizes an epitope of an isoform of HSP90, GRP94 selectively expressed on cancer cell surface [37]) at 4 °C for 1 h, then incubated respectively with Allophycocyanin (APC) AffiniPure F (ab’)_2_ Fragment Goat Anti-Mouse IgG (H + L) or R-Phycoerythrin AffiniPure F (ab’)_2_ Fragment Goat Anti-Human IgG, Fcγ fragment from Jackson ImmunoResearch Laboratories, Inc. USA. Isotype-matched IgG antibodies were used as controls**.** The following PE-conjugated rabbit mAbs (1:50) from Cell Signaling were used: mAb for CRT (D3E6) (for Fig. 4c 24h post IR staining only), mAb for HSP90 (C45G5) (for Fig. 4d 24h post IR staining only) and mAb IgG Isotype Control (DA1E) as a specificity control for both rabbit anti- CRT or -HSP90 mAbs (Fig. 4c, d). Each sample was analyzed by BD Accuri™ C6 Flow Cytometer (BD Bioscience) and FlowJo software.

### Western blot analysis

Cells were plated in 6-well plates at a density of 5 x 10^4^ cells in 2 mL of the culture medium and grown overnight. The cells were treated with 0.15/1 μM DSF/Cu for 24 h, then irradiated with 12Gy and cultured for additional 24 h before being lyzed. The cell lysates were used for detection of cleaved PARP by western blot using rabbit mAb ((Asp214) (D64E10),1:1000, Cell Signaling), and β-actin rabbit mAb ((13E5), 1:1000, Cell Signaling) for a protein loading control, as described [34].

### Detection of intracellular and extracellular ATP

Intracellular ATP was stained with the ATP-sensitive fluorochrome quinacrine [38]*.* Cells (5 × 10^4^) were incubated with 10 μM quinacrine dihydrochloride (Sigma) in 100 μL PBS at 37 °C for 1 h. Then the cells were washed 4 time with PBS and resuspended in 100 μL for flow analysis. Fluorescence was detected by flow cytometer at 510–530 nm with excitation at 488 nm [39]. A decreased level of intracellular ATP reflects an increase of extracellular ATP release. To confirm it, we measured quantitatively extracellular released ATP by testing some of the collected cell culture supernatants (by centrifugation at 2000 rpm, 10 min, 4 °C, MICROCL 17R, Thermo Scientific) with ATP Bioluminescence Assay Kit HS II (Roche, Mannheim, Germany) per manufacturer’s instructions, with the Synergy 2 Multi-Detection Microplate Reader (BioTek, Winooski, USA).

### Quantitation of extracellular HMGB1

The culture supernatants of cells were collected for detection of HMGB1 release at the same time when the cells were harvested for flow analysis. The concentrations of HMGB1 in undiluted supernatants were measured using HMGB1 ELISA Kit according to the manufacturer’s instructions (ABIN511375, IBL America, MN, USA).

### Statistical analysis

Data were analyzed by SPSS version 19.0 (SPSS Inc., Chicago, IL, USA) and determined by one-way ANOVA. The results were obtained in 2–3 independent experiments. Differences between groups were considered significant when *p* < 0.05.

## Results

### Identification and flow sorting of BCSCs from breast cancer cell lines

**Fig. 1:**
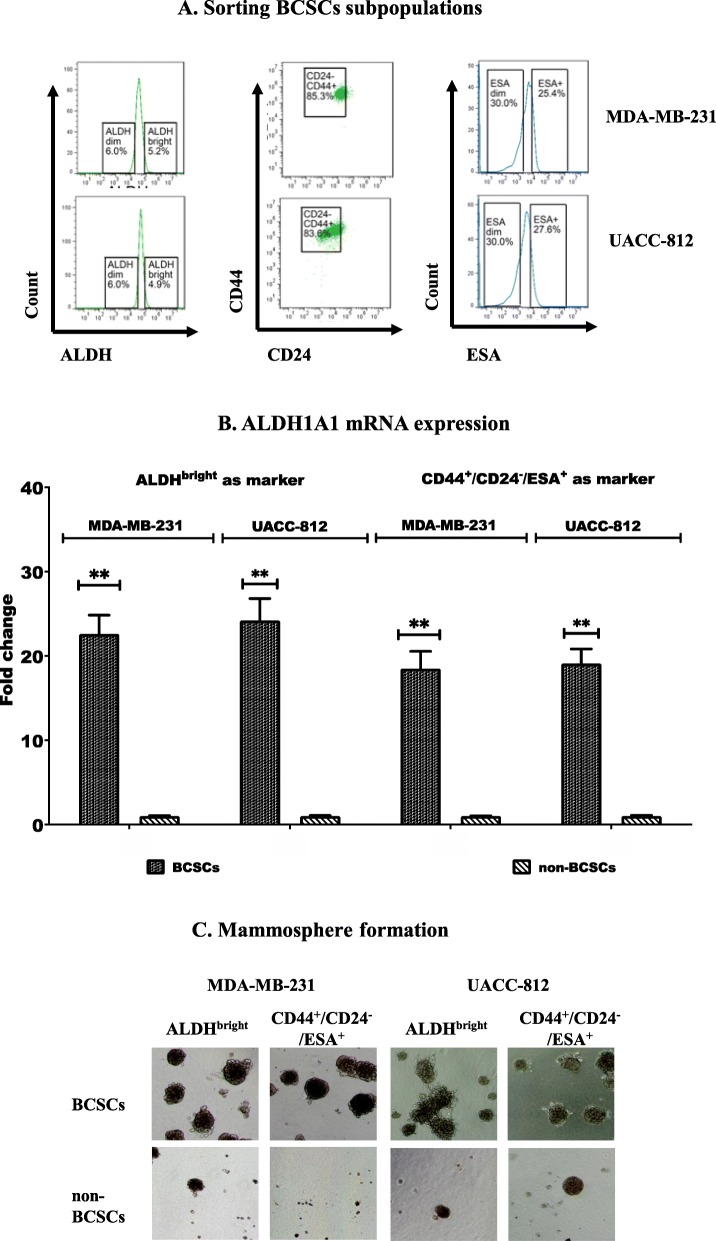
Identification and sorting of BCSCs using flow cytometry from the human breast cell lines. (**a**) Sorting of BCSCs identified as either ALDH^bright^ or CD44^+^/CD24^−^/ESA^+^ cells from MDA-MB-231 and UACC-812 cell lines (**b**) Sorted ALDH^bright^ as well as CD44^+^/CD24^−^/ESA^+^ BCSCs expressed greater levels of ALDH1A1 mRNA than in non-BCSCs (**c**) Mammosphere formation from sorted BSCSs and non-BSCSs

ALDH^bright^ and CD44^+^/CD24^−^/ESA^+^ tumor cells, previously shown to have BCSC properties, were isolated from human MDA-MB-231 and UACC-812 breast cancer cell lines by FACS (Fig. 1a). BCSCs in MDA-MD-231 cells consisted of 5.2% ALDH^bright^ and 21.6% CD44^+^/CD24^-^/ESA^+^ cells (sorted cells were 85.3% CD44^+^/CD24^-^ of which 25.4% were ESA^+^, therefore % of CD44^+^/CD24^-^/ESA^+^ cells was 85.3% x 25.4% = 21.6%). The UACC-812 cell line contained 4.9% ALDH^bright^ and 23.1% CD44^+^/CD24^-^/ESA^+^ cells (sorted cells were 83.6% CD44^+^/CD24^-^ of which 27.6% were ESA^+^, therefore % of CD44^+^/CD24^-^/ESA^+^ cells was 83.6% x 27.6% = 23.1%). ALDH^dim^ and ESA^−^ cells in MDA-MB-231 and UACC-812 cell lines, which were considered as non-BCSCs, were also sorted from these cell lines. In addition, we did qRT-PCR on sorted BCSCs vs non-BCSCs for mRNA expression of ALDH1A1 which is one of the main contributors to ALDH activity detected by ALDEFLUOR [40]. The data were consistent with ALDEFLUOR assay by which we identified and sorted ALDH^bright^/ ALDH^dim^ cells, i.e., ALDH^bright^ cells expressed higher levels (18–25 fold increase) of ALDH1A1 mRNA than that in ALDH^dim^ cells (Fig. 1b). The mammosphere formation abilities of the sorted cell populations isolated from both cell lines were determined. The results indicate that BCSCs sorted from the two human breast cancer cell lines had a more pronounced ability to form mammospheres, a key functional property of BCSCs, than the non-BCSCs sorted from them **(**Fig. 1c).

### IR triggered a lower level of ICD in BCSCs than in non-BCSCs

**Fig. 2:**
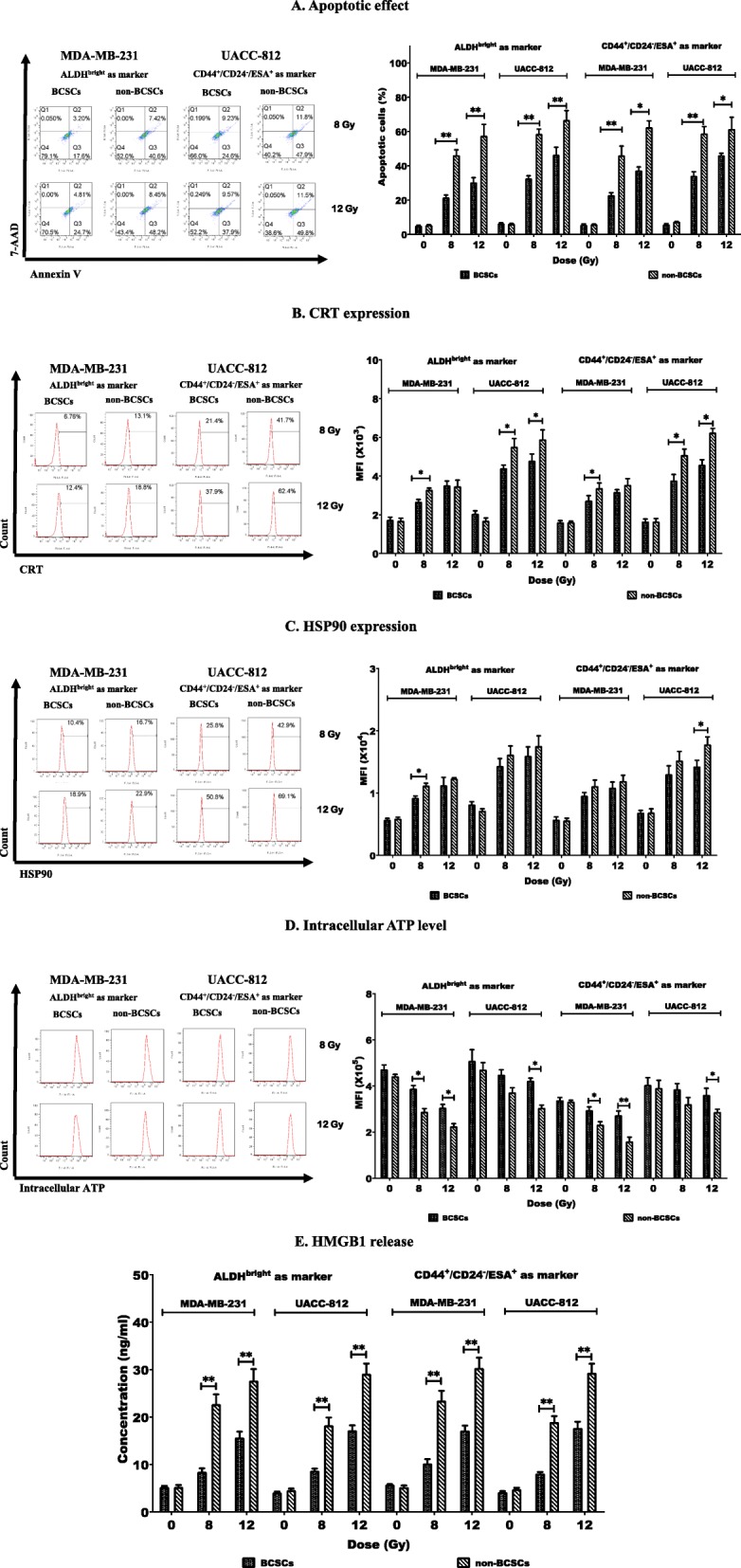
Radiation induced a low level of ICD in BCSCs than in non-BCSCs. Sorted cells were treated with IR at 8 or 12 Gy and cultured. Then cells and/or culture supernatants were collected at indicated times post IR and used for analyses of ICD by flow cytometry and ELISA. (**a**) Apoptotic cells were quantitated 24 h post-IR using AnnexinV/7-AAD staining (left panel). Percentages of apoptotic cells are indicated (right panel). (**b**) Percentages of cells expressing CRT were detected 8 h post IR (left panel) and their mean fluorescence intensity (MFI) values are shown (right panel). (**c**) Percentages of cells expressing HSP90 were detected 8 h post IR (left panel) and their MFI values are shown (right panel). (**d**) Levels of intracellular ATP were measured 1 h post-IR (left panel) and their MFI values are shown (right panel). (**e**) Concentrations of HMGB1 release were determined in supernatants collected at 24 h post-IR. *indicates *p* < 0.05, **indicates *p* < 0.01

Since it is generally considered that BCSCs existing in solid tumors contribute to RT resistance [6, 41, 42], the conditions under which IR can induce ICD of BCSC was investigated using sorted ALDH^bright^ and CD44^+^/CD24^−^/ESA^+^ BCSCs exposed to 8 or 12 Gy. These cells were then monitored for apoptosis and the above-mentioned parameters of IDC-mediating DAMPs. Apoptosis was induced by 8 Gy and 12 Gy IR in BCSCs and non-BCSCs of both breast cancer cell lines as showed in Fig. 2a. IR (8Gy) induced apoptosis in both sorted BCSC populations, which ranged from 21.5 ± 1.5% to 34.1 ± 2.4%, while the range of apoptotic cells in non-BCSCs was higher at 46.1 ± 5.4% to 58.9 ± 4.0. The percentages of apoptotic cells following IR (12 Gy) ranged from 30.3 ± 2.8% to 46.4 ± 4.4% in BCSCs and 57.5 ± 6.7% to 66.7 ± 5.5% in non-BCSCs. Therefore, regardless of whether 8 Gy or 12 Gy was employed, the differences of the lower rates of apoptosis of BCSCs compared to that of non-BCSCs were significant (*p* < 0.05 or *p* < 0.01) (Fig. 2a).

In contrast to apoptosis, the index of RT-induced ICD relative to DAMPs varied with the breast cancer cell line tested, defined BCSC phenotype and IR dosage. CRT expression was significantly higher on the surface of the sorted non-BCSCs compared to BCSCs of UACC-812 cell line, regardless of whether they were sorted as ALDH^bright^ or CD44^+^/CD24^−^/ESA^+^ BCSCs and whether treated with 8 Gy or 12 Gy IR (*p* < 0.05 or *p* < 0.01). In regard to MDA-MB-231 cells, significant differences between the CRT expression of non-BCSCs and either sorted BCSC phenotype were noted at 8 Gy IR (*p* < 0.05) but not 12Gy (Fig. 2b). Regarding HSP90 cell surface expression, the findings showed more diversity. MDA-MB-231 ALDH^bright^ BCSCs showed a significantly lower HSP90 expression than non-BCSCs at 8 Gy but not 12 Gy, whereas UACC-812 ALDH^bright^ BCSCs showed no differences regardless of IR dosage. However, UACC-812 CD44^+^/CD24^−^/ESA^+^ BCSC showed a difference in HSP90 expression at 12 Gy but not 8 Gy IR (*p* < 0.05), with a trend of more increasing HSP90 expression in non-BCSCs than BCSCs in responding to IR at 8 Gy or 12 Gy being noticeable (Fig. 2c). The third ICD parameter monitored was intracellular ATP, which decreases with ICD. As shown in Fig. 2d, intracellular ATP levels were significantly decreased, in sorted non-BCSCs compared with sorted ALDH^bright^ or CD44^+^/CD24^−^/ESA^+^ BCSCs obtained from MDA-MB-231 cells after 8 Gy or 12 Gy IR (*p* < 0.05 or *p* < 0.01), but there was a difference in UACC-812-derived non-BCSC populations compared to both types of sorted BCSC populations only after 12 Gy IR (*p* < 0.05). Nevertheless, a trend of more decreased intracellular ATP in non-BCSCs than BCSCs in responding to IR at 8 Gy or 12 Gy was noted.

At either 8 Gy or 12 Gy IR, the fourth ICD parameter monitored, HMGB1 release, showed significant differences between the two types of sorted BCSC phenotypes and non-BCSCs derived from both cell lines (*p* < 0.05 or *p* < 0.01) (Fig. 2e).

These data demonstrated that IR induced less apoptosis, lower levels of cell surface expression of CRT and HSP90 and lower levels of ATP and HMGB1 release in sorted BCSCs of either phenotype than in sorted non-BCSCs, which indicates that IR induced lower levels of ICD in BCSCs than in non-BCSCs. A summary of these findings is shown in Table 1.

**Table 1:** ICD hall markers showed in BCSCs vs non-BCSCs after irradiation

	MDA-MB-231				UACC-812			
	ALDH^bright^ as marker		CD44+/CD24−/ESA+ as marker		ALDH^bright^ as marker		CD44+/CD24−/ESA+ as marker	
	8 Gy	12 Gy	8 Gy	12 Gy	8 Gy	12 Gy	8 Gy	12 Gy
Apoptosis	↓↓	↓↓	↓↓	↓	↓↓	↓↓	↓↓	↓
CRT	↓	–	↓	–	↓	↓	↓	↓
HSP90	↓	–	–	–	–	–	–	↓
ATP	↑	↑	↑	↑↑	–	↑	–	↑
HMGB1	↓↓	↓↓	↓↓	↓↓	↓↓	↓↓	↓↓	↓↓

↑ (down) and↓(up). P < 0.05, ↑↑ and ↓↓ P < 0.01, – not significant (BCSCs vs non-BCSCs)

### DSF/Cu induced a similar extent of IDC in both BCSCs and non-BCSCs

**Fig. 3:**
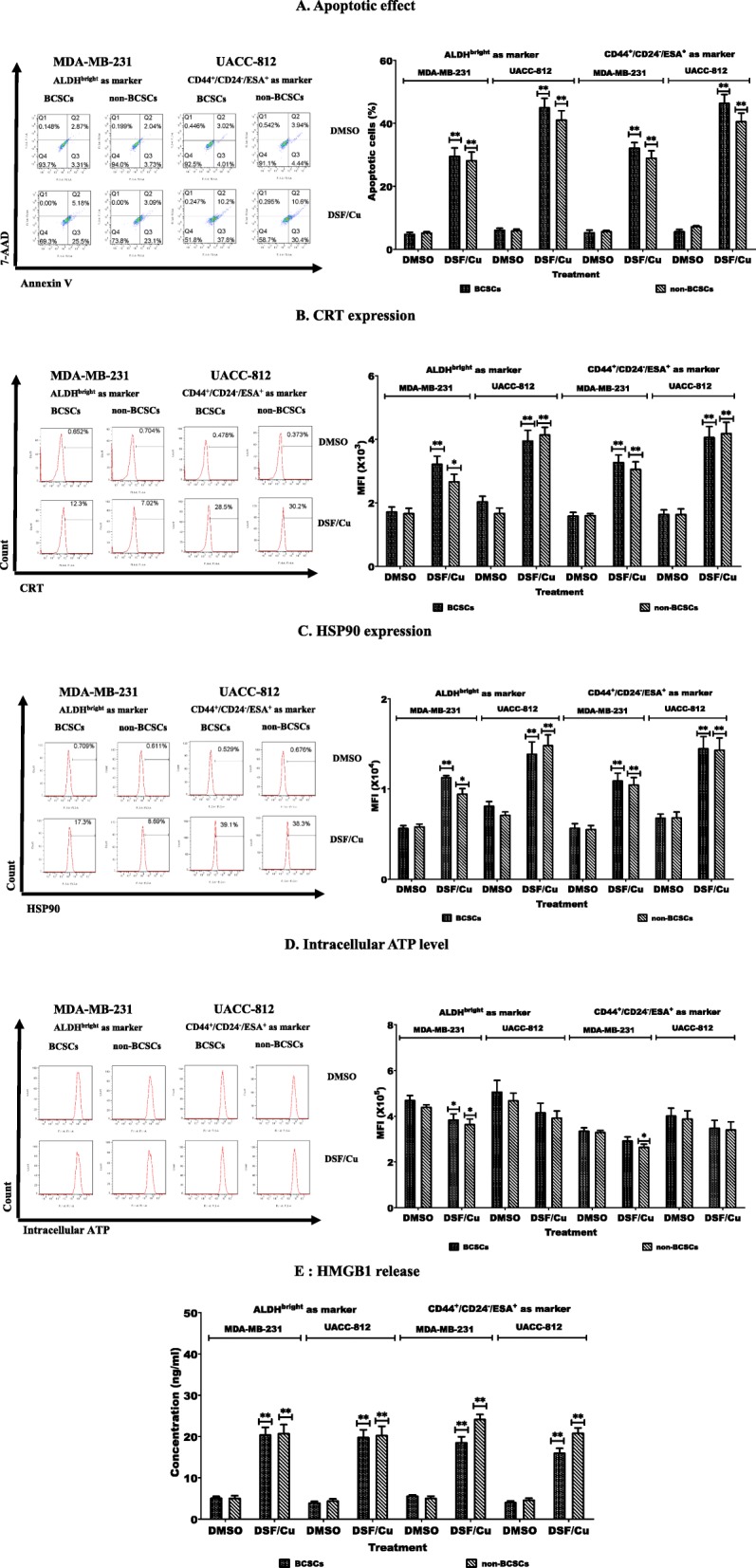
DSF/Cu induced a similar extent of IDC in both BCSCs and non-BCSCs. Sorted cells were treated with (0.15/1 μM) DSF/Cu for 24 h and then cells and supernatants were analyzed for ICD. (**a**) Apoptotic cells were quantitated (left panel). Percentage of apoptotic cells are indicated (right panel). (**b**) Percentages of cells expressing CRT (left panel) and their mean MFI values are indicated (right panel). (**c**) Percentages of cells expressing HSP90 (left panel) and their MFI values (right panel). (**d**) Levels of intracellular ATP were measured (left panel) and their MFI values are shown (right panel). (**e**) Concentrations of HMGB1 release were determined in supernatants. *indicates *p* < 0.05, **indicates *p* < 0.01 (DSF/Cu treated vs DMSO treated cells)

CSCs are resistant to most standard radiotherapies as well as chemotherapies. Our previous studies revealed that DSF/Cu can preferentially eliminate BCSCs and pancreatic CSCs in vitro and in vivo in the context of IR or chemoradiation [6, 17]. However, the ability of DSF/Cu in the induction of ICD of BCSC and non-BCSC populations has not been investigated to date. Therefore, the effect of DSF/Cu and the control diluent vehicle, DMSO, on ICD of both sorted phenotype BCSC populations as well as non-BCSCs was initially assessed. Apoptosis was detected in all four cell populations derived from MDA-MB-231 and UACC-812 cells, as showed in Fig. 3a. DSF/Cu induced apoptosis in both sorted BCSCs and non-BCSCs compared to DMSO (*p* < 0.05), and there were no obvious differences on apoptotic ratios in sorted BCSCs and non-BCSCs isolated from MDA-MB-231 and UACC-812 cell lines. DSF/Cu treatment increased CRT (Fig. 3b) and HSP90 (Fig. 3c) expression on the surface of all four cell populations analyzed (*p* < 0.05 or *p* < 0.01). Intracellular ATP levels were decreased in the non-BCSCs and two phenotypic distinct BCSCs sorted from MDA-MB-231 cell line (*p* < 0.05 or *p* < 0.01) and a trend of decreased intracellular ATP in BCSCs and non-BCSCs sorted from UACC-812 cell line was noted (Fig. 3d). HMGB1 release was significant increased in all the DSF/Cu-treated sorted cells (*p* < 0.01) (Fig. 3e).

### DSF/Cu rendered BCSCs as sensitive as non-BCSCs to IR-induced ICD

**Fig. 4:**
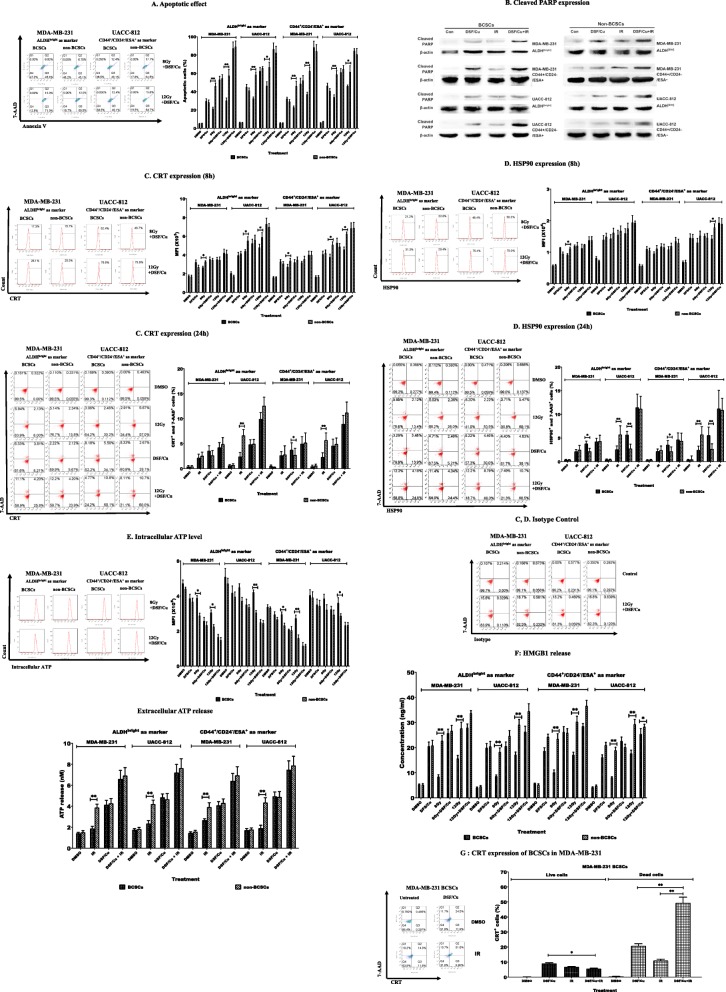
DSF/Cu rendered BCSCs as sensitive as non-BCSCs to IR-induced ICD. Sorted sells were pretreated with DSF/Cu (0.15/1 μM) for 24 h, then DSF/Cu containing medium was removed and replaced with fresh culture medium and then irradiated at 8 or 12 Gy and cultured. The cells and/or culture supernatants were collected at indicated times post IR and used for analyses of ICD. (**a**) Apoptotic cells were quantitated 24 h post-IR (left panel). Percentages of apoptotic cells are indicated (right panel). (**b**) Cleaved PARP, an indicator of apoptosis, was detected in both BCSCs and non-BCSCs treated by DSF/Cu and IR. (**c**) Percentages of cells expressing CRT were detected 8 h post IR (left panel) and their mean fluorescence intensity (MFI) values are shown (right panel); Percentages of 7AAD^+^ cells expressing CRT were detected 24 h post IR (left panel) and means ± SD of 7AAD^+^CRT^+^ cells (%) are shown (right panel). (**d**) Percentages of cells expressing HSP90 were detected 8 h post IR (left panel) and their MFI values are shown (right panel); Percentages of 7AAD^+^ cells expressing HSP90 were detected 24 h post IR (left panel) and means ± SD of 7AAD^+^HSP90^+^ cells (%) are shown (right panel), PE-conjugated rabbit isotype control mAb was simultaneously stained cells with PE-conjugated rabbit mAb for CRT and HSP90 24 h post IR-negative control results shown as C, D isotype control. (**e**) Levels of intracellular ATP were measured 1 h post-IR (left panel) and their MFI values are shown (right panel); Levels of extracellular ATP release were measured 24 h post-IR. (**g**) Concentrations of HMGB1 release were determined in supernatants collected at 24 h post-IR. (**g**) CRT expression was quantitated in dead (7-AAD^+^) and live (7-AAD^−^) MDA-MB-231 BCSCs. *indicates *p* < 0.05, **indicates *p* < 0.01

To determine whether DSF/Cu can enhance the sensitivity of BCSCs to IR-induced ICD, BCSCs and non-BCSCs sorted from MDA-MB-231 and UACC-812 cell lines were irradiated following DSF/Cu treatment and monitored for apoptosis and the four parameters of ICD-mediating DAMPs previously detailed. DSF/Cu in combination with IR (8 Gy or 12 Gy) resulted in increased ICD including apoptosis, CRT and HSP90 expression, and ATP and HMGB1 release in all four sorted cell populations **(**Fig. 4a-g).

The significant differences in apoptosis between BCSCs and non-BCSCs in responding to IR only (8 or 12 Gy) previously noted disappeared when they were treated with DSF/Cu prior to IR. In other words, DSF/Cu made BCSCs equally sensitive to IR-induced apoptosis as non-BCSCs (Fig. 4a). The apoptosis of cells was confirmed by detecting cleaved PARP, an indicator of apoptosis, in western blotting (Fig. 4b) In addition, comparing to 8 or 12 Gy IR alone, prior treatment of the two types of BCSC populations sorted from MDA-MB-231 and UACC-812 cell line with DSF/Cu at 8 or 24h showed increased cell surface CRT expression compared to sorted non-BCSCs from these cell lines (Fig. 4c); a similar finding in HSP90 cell surface expression was obtained in ALDH^bright^ BCSCs from MDA-MB-231 cell line and CD44^+^/CD24^−^/ESA^+^ BCSCs from UCAA-812 cell line to 8 and 12 Gy IR, respectively. The staining was specific as isotype controls was negative in dead/dying cells (Fig. 4c, d). Moreover, BCSCs defined either by ALDH^bright^ or CD44^+^/CD24^−^/ESA^+^ had decreased intracellular ATP, reflecting increased extracellular ATP and increased ATP release in cell supernatants to 8 or 12Gy IR (Fig. 4e); lastly, all four BCSC populations had increased HMGB1 release to 8 and 12 Gy IR (Fig. 4f).

Furthermore, to determine whether CRT is indeed expressed by DSF/Cu and IR-induced 7-AAD^+^ dying cells, ALDH^bright^ BCSCs sorted from MDA-MB-231cell line were analyzed with 7-AAD^+^ dying and 7-AAD^−^ live cells for CRT expression. Following treatment with DSF/Cu and IR, 49.4 ± 3.8% were 7-AAD^+^CRT^+^, which is significantly higher than that in either DSF/Cu or IR treated ALDH^bright^ BCSCs (*p* < 0.001) (Fig. 4g). On the other hand, only 5.6 ± 0.4% of 7-AAD^−^ cells were CRT^+^ (Fig. 4g). The data indicate that combination of DSF/Cu and IR at the indicated doses induced ICD in ~ 50% of BCSCs compared to ~ 10% of that in BCSCs treated by IR alone. These data suggested that DSF/Cu rendered IR-resistant BCSCs as sensitive as non-BCSC to IR-induced ICD.

### ROS generation and IRE1α/XBP1 axis are partially responsible for increased IR-induced ICD of BCSCs by DSF/Cu

**Fig. 5:**
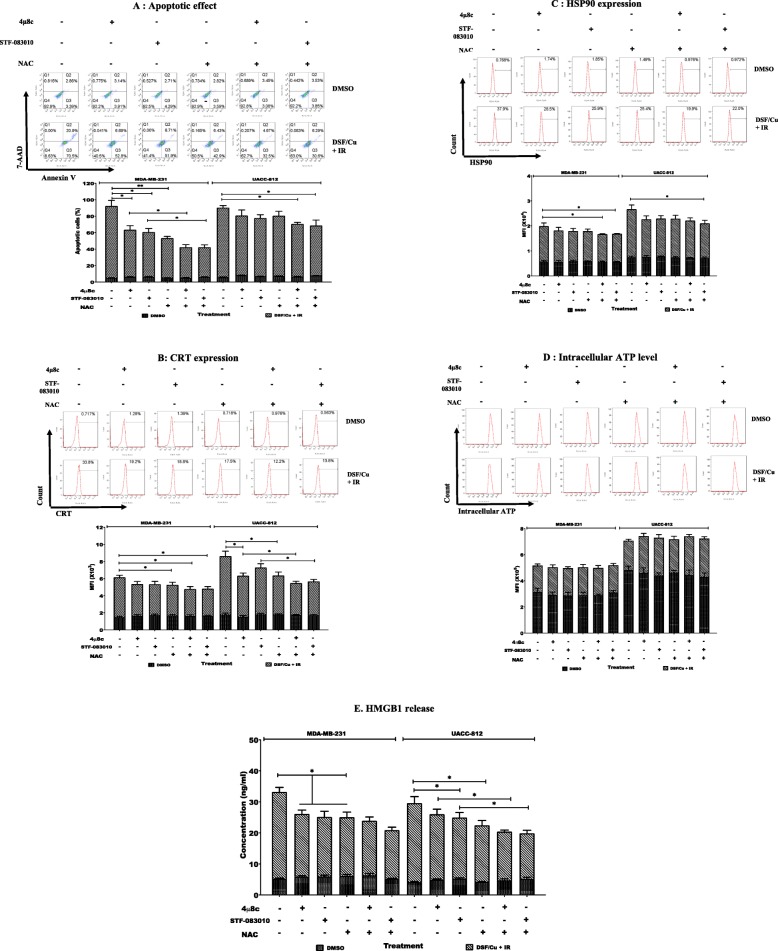
Blockade of ROS and IRE1α/XBP1s axis inhibited ICD induced by DSF/Cu and irradiation in BCSCs. Sorted BCSCs were pretreated with 10 μM 4u8c, 10 μM STF-083010 or 10 mM NAC for 1 h. The inhibitor(s) containing medium was replaced with fresh culture medium with DSF/Cu (0.15/1 μM) and cultured for 24 h. Then DSF/Cu containing medium was removed and replaced with fresh culture medium and then irradiated at 12 Gy and cultured. The cells and/or culture supernatants were collected at indicated times post IR and used for analyses of ICD. (**a**) Apoptotic cells were quantitated 24 h post-IR (top panel). Percentages of apoptotic cells are indicated (bottom panel). (**b**) Percentages of cells expressing CRT were detected 8 h post IR (top panel) and their mean fluorescence intensity (MFI) values are shown (bottom panel). (**c**) Percentages of cells expressing HSP90 were detected 8 h post IR (top panel) and their MFI values are shown (bottom panel). (**d**) Levels of intracellular ATP were measured 1 h post-IR (top panel) and their MFI values are shown (bottom panel). (**e**) Concentrations of HMGB1 release were determined in supernatants collected at 24 h post-IR. *indicates *p* < 0.05, **indicates *p* < 0.01. These data demonstrate that blocking ROS generation and IRE1α/XBP1s signaling pathway partially abolished elevated ICD of BCSCs by IR and DSF/Cu and implies that both pathways are involved in the enhanced immunogenic apoptosis of BCSCs induced by this combinatorial approach

It is known that DSF/Cu generates ROS [15]. Our recent work demonstrated DSF/Cu induces strong ER stress through activation of the IRE1α/XBP1 axis of the unfolded protein response (UPR) [16]. These findings, especially the latter, led to the hypothesis that DSF/Cu could induce IDC since ER stress and ROS production are key players of the intracellular signaling pathways that govern ICD [27]. To this end, the functional importance of ROS in the enhanced ICD of sorted BCSCs sensitized to IR by DSF/Cu was examined by monitoring apoptosis and the four DAMPs associated with ICD in BCSCs pretreated with the ROS scavenger, N-acetyl cysteine (NAC) prior to DSF/Cu exposure and IR. Concurrently, the role of the IRE1α/XBP1 axis in ICD of BCSCs induced by DSF/Cu and IR was examined. When BCSCs were pretreated with NAC or the IRE1α/XBP1 pathway inhibitors STF-083010 or 4μ8c, the elevated apoptosis induced by pretreatment with DSF/Cu followed by IR 12Gy for 24 h was suppressed (*p* < 0.05 or *p* < 0.01 (Fig. 5a). Moreover, combinations of NAC with either STF-083010 or 4μ8c showed more pronounced inhibition of DSF/Cu and IR induced apoptosis of BCSCs compared to either single inhibitor treatment of the MDA-MB-231 cell line (*p* < 0.05) (Fig. 5a). Apoptosis of UACC-812 BCSCs was suppressed only by combinative pretreatment with STF-083010 and NAC or 4μ8c and NAC (*p* < 0.05) (Fig. 5a). CRT surface expression of MDA-MB-231 BCSCs was suppressed when cells were pretreated with NAC alone or NAC combined with STF-083010 or 4μ8c (*p* < 0.05) (Fig. 5b). A similar inhibition pattern of CRT surface expression on UACC-812 BCSCs was evidenced except 4μ8c inhibited CRT expression as a single agent (*p* < 0.05) (Fig. 5b). For all the BCSCs examined, only the combination of STF-083010 and NAC or 4μ8c and NAC showed suppressive function on HSP90 surface expression (*p* < 0.05) (Fig. 5c). However, intracellular ATP levels showed no changes when BCSCs were pretreated with any single inhibitor or inhibitor combinations (Fig. 5d), whereas all the agents individually suppressed HMGB1 release in all the BCSCs populations studied (*p* < 0.05) (Fig. 5e) and combination of NAC with either STF-083010 or 4μ8c showed stronger suppression of HMGB1 release compared to single inhibitor pretreatment in UCAA-812 cell line (*p* < 0.05) (Fig. 5e).

## Discussion

BCSCs are resistant to chemotherapy and radiation [2, 6, 41, 43] and recent studies demonstrated that BCSCs escape from innate and adaptive immune responses, which suggests that BCSCs are resistant to immunotherapy as well [44–48]. These findings underscore the importance of developing novel approaches that make BCSCs as sensitive as non-BCSCs to conventional therapies and immunotherapy in order to develop successful and curative treatments for breast cancer patients. It is known that IR can trigger ICD of breast and other cancer cells [23], but clinically, RT is often inadequate in generating sufficient immuno-stmularoty signals to optimally activate innate and adaptive immune responses. Thus, IR may induce a level of ICD in BCSCs that is insufficient to generate an effective anti-tumor immune response. We hypothesized that DSF/Cu is an ideal agent to induce ICD for the following reasons: i) it preferentially targets CSCs [17], ii) induces ROS [15], and iii) induces ER stress and autophagic apoptosis [16]. Both ER stress and ROS are key players of intracellular signaling pathways that govern ICD [27], and in addition, autophagy can trigger ICD [26]. Therefore, we investigated the ability of DSF/Cu to induce IDC of BCSCs and non-BCSCs and the possibility for DSF/Cu to render radiation-resistant BCSCs as sensitive as radiation-sensitive-non-BCSCs.

Compared to non-BCSCs, IR at tested single doses (8 and 12 Gy) only induced a much lower level of ICD in BCSCs than non-BCSCs. The BCSCs were identified in both MDA-MB-231 (triple negative) and UACC-812 (ER^+^, HER^amp^) cell lines [49] by either high ALDH activity or expression of the cell surface phenotype CD44^+^/CD24^−^/ESA^+^. As expected, DSF/Cu was able to induce a similar extent of ICD in both BCSCs and non-BCSCs. Pretreatment with a low dose of DSF (0.15 μM) and a physiological level of CuCl_2_(1 μM) rendered IR-resistant BCSCs as sensitive as non-BCSC to IR-induced ICD. All the IDC parameters studied, the percentage of apoptotic cells, CRT and HSP90 cell surface expression, and release of HMGB1 and ATP in BCSCs were increased by DSF/Cu and IR treatment. ROS and IRE1α/XBP1 axis also appear to be involved in DSF/Cu and IR-induced ICD of BCSCs, since ROS scavenger and IRE1α/XBP1 pathway inhibitors combined partially blocked apoptosis, CRT and HSP90 cell surface expression and HMGB1 release, but not ATP levels in these treated cells. One can conclude, therefore, that in addition to enhanced ROS production and IRE1α/XBP1 axis activation, DSF/Cu and IR-induced ICD of BCSCs should involve other mechanisms.

To our knowledge, our study demonstrates, for the first time, that IR triggered a lower level of ICD in BCSCs than in non-BCSCs using the in vitro characteristic parameters of apoptosis and ICD-mediating DAMPs. Consistent with the fact that BCSCs are IR-resistant, BCSCs are also resistant to IR-induced ICD. Therefore, by taking advantage of using DSF/Cu pretreatment, we could induce the same extent of ICD in both BCSCs and non-BCSCs by IR. This is the first study aiming at induction of ICD in radiation-resistant BCSCs by repurposing DSF. The results derived from this in vitro-based study has prompted our laboratory to initiate an ongoing in vivo*-*based study using preclinical tumor models to investigate the potential of IR and DSF/Cu-induced ICD of BCSCs and non-BCSCs as a potent vaccine to activate the host’s immune system to generate robust and durable anti-tumor immune responses against primary breast tumors as well as distant metastasis. Additionally, our finding is fundamental to the opening of a new research area aimed at exploring the possibility of using BCSCs as an immunogen in breast cancer vaccines to induce effective systemic immune responses against not only differentiated/ differentiating breast cancer cells but also BCSCs, the root cause of breast cancer formation, progression and metastasis.

## Conclusions

In this study, we identified that BCSCs are resistant to IR-induced ICD. However, we found that DSF/Cu rendered IR-resistant BCSCs as sensitive as non-BCSCs to IR-induced ICD. This may due partially to DSF/Cu induced ROS production and IRE1α/XBP1 axis activation. Our finding is fundamental to the opening of a new research area aimed at exploring the possibility of using BCSCs as the immunogen in a IR-based breast cancer vaccine.

## Abbreviations

ALDH: aldehyde dehydrogenase
APCs: Antigen presenting cells
ATCC: American Type Culture Collection
ATP: Adenosine triphosphate
BCSCs: Breast cancer stem cells
CRT: Calreticulin
CSCs: Cancer stem cells
DAMPs: Damage-associated molecular pattern molecules
deDTC: diethyldithiocarbamate
DSF/Cu: Disulfiram/cooper
ER: endoplasmic reticulum
Gy: Gray
HMGB1: High mobility group protein B1
HSPs: Heat shock proteins
ICD: Immunogenic cell death
IR: Irradiation
IRE1α: Inositol requiring-enzyme 1 alpha
ROS: Reactive oxygen species
RT: Radiation therapy
XBP1: X-box-binding protein 1
XBP1s: Spliced/active form of XBP1
