# Correction to: Induction of immunogenic cell death in radiation-resistant breast cancer stem cells by repurposing anti-alcoholism drug disulfiram

**DOI:** 10.1186/s12964-020-00567-0

**Published:** 2020-04-02

**Authors:** Ting Sun, Wei Yang, Sneh M. Toprani, Wei Guo, Lile He, Albert B. DeLeo, Soldano Ferrone, Gong Zhang, Enwen Wang, Zunwen Lin, Pan Hu, Xinhui Wang

**Affiliations:** 1 Division of Surgical Oncology, Department of Surgery, Massachusetts General Hospital, Harvard Medical School, Boston, USA; 2 Neurosurgery and Brain and Nerve Research Laboratory, The First Affiliated Hospital of Soochow University, Suzhou, Jiangsu China; 3 John B. Little Center for Radiation Sciences, Harvard T.H. Chan School of Public Health, Boston, USA; 4 State Key Laboratory of Radiation Medicine and Protection, School of Radiation Medicine and Protection and Collaborative Innovation Center of Radiation Medicine of Jiangsu Higher Education Institutions, Soochow University, Suzhou, China; 5 Department of Orthopaedic Surgery, Massachusetts General Hospital, Harvard Medical School, Boston, USA

**Correction to: Cell Commun Signal (2020) 18:36**

**https://doi.org/10.1186/s12964-019-0507-3**

Following publication of the original article [1], the authors reported an error in the section **Mammosphere formation assay**. 5 mg/mL insulin should be 5 μg/mL insulin. The second sentence should therefore read as follows:

The medium contained 32% MethoCult medium, 20% MammoCult basal human medium with a final concentration of 2% MammoCult proliferation supplements and 48% DMEM supplemented with final concentrations of 100 pg/mL EGF, 50 ng/mL bFGF, 5 ng/mL stem cell factor, 1 μM hydrocortisone, and 5 μg/mL insulin, all obtained from STEMCELL Technologies.

The authors apologise for this error.
